# Shifting Perspectives on the Challenges of Shared Decision Making in Mental Health Care

**DOI:** 10.1007/s10597-023-01170-6

**Published:** 2023-08-07

**Authors:** Doris Verwijmeren, Koen P. Grootens

**Affiliations:** 1 Tranzo, Tilburg School of Social and Behavioral Sciences, Tilburg University, Postbus 90153, 5000 LE Tilburg, The Netherlands; 2 Reinier van Arkel Mental Health Institute, ‘s-Hertogenbosch, The Netherlands

**Keywords:** Shared decision making, Patient-centred care, Mental health, Review, Recovery-oriented health care

## Abstract

Although shared decision making (SDM) has become the most preferable way in doctor–patient communication, it is not fully implemented in mental health care likely due to the complex nature of psychiatric syndromes and treatments. In this review we provide a systematic overview of all perceived and reported barriers to SDM in the literature, acknowledging field-specific challenges, and offering perspectives to promote its wider use. We conducted a systematic search of the wider literature in different databases and included all publications mentioning specified barriers to SDM in psychiatric care. Relevant data and opinions were categorised into micro-, meso- and macro-level themes and put into clinical perspective. We derived 20 barriers to SDM from 100 studies and reports. Eight were on micro-level care delivery, seven involved meso-level issues, five concerned macro-level themes. The multitude of perceived and actual barriers to SDM underline the challenges its implementation poses in mental health care, some of which can be resolved while others are inherent to the nature of the care, with its long-term relationships, complex dynamics, and social consequences, all requiring a flexible approach. We present four perspectives to help change views on the potential of SDM in mental health care.

## Introduction

Shared decision making (SDM) has become the most preferable way in today’s doctor–patient communication (Slade, 2017). SDM can be defined as an interactive process between at least two expert parties, that is service user, sometimes accompanied by a next of kin or caregiver, and service provider, where information and opinions are shared and provider responsibilities and recommendations and patient preferences and goals are discussed (Zisman-Ilani et al., 2021b). Most authors describe SDM as an intermediate approach between the paternalistic “doctor-decides-alone” model and the informed-choice “doctor-informs, patient-decides” paradigm (Hamann et al., 2003).

In the literature one will find many different interpretations of SDM (Stiggelbout et al., 2015), where the initial concept of engaging patients in health care decisions has evolved to include many new factors from the micro level of the consultation room up to the macro level of society at large. Still, the international consensus is that SDM should be implemented across the field of medicine (Deegan, 2010; Huang et al., 2020a; Lovell et al., 2018; Slade, 2017). Many potential and achieved benefits of SDM are described, such as increased involvement, reduced stigma, and improved patient satisfaction (Bradley & Green, 2017; Brennan et al., 2019; Duncan et al., 2010; Hamann et al., 2006; Hayes et al., 2019a; Langer & Jensen-Doss, 2016; Loh et al., 2007; Nott et al., 2018), and even improved quality of care and more effective service delivery with possible economic benefits are mentioned (Jorgensen & Rendtorff, 2018). It needs to be noted, though, that to date no clear effect on adherence outcomes and symptom reduction has been demonstrated (Aoki et al., 2022).

The principles of SDM seem to fit in perfectly with the pragmatic solutions and patient-specific decisions that are so often needed in mental health care especially when treating severe disorders, where finetuned communication skills, patient empowerment, and personalised recovery-oriented care are vital elements. However, previous studies have shown that, as yet, SDM is not widely implemented in standard psychiatric care, with many single barriers to its implementation being described (Hamann & Heres, 2014; Hopwood, 2020; Huang et al., 2020a; Lovell et al., 2018). At this point, there is no clear overview of the exact nature or span of the obstacles to SDM that are perceived in the field.

Perceived barriers in combination with the broad concept SDM has become may then hamper the implementation of SDM in mental health practice. To provide an overview of the difficulties SDM poses we reviewed the literature and categorised the obstacles reported thematically. Since hurdles in implementation processes may stem from objective observations as well as subjectively experienced difficulties, we purposely restrained from using strict methodological inclusion criteria and looked both at expert opinion and empirical data. Finally, acknowledging practical challenges, we offer some fundamental perspectives on SDM to try and promote its wider implementation in mental health care.

## Methods

**Fig. 1:**
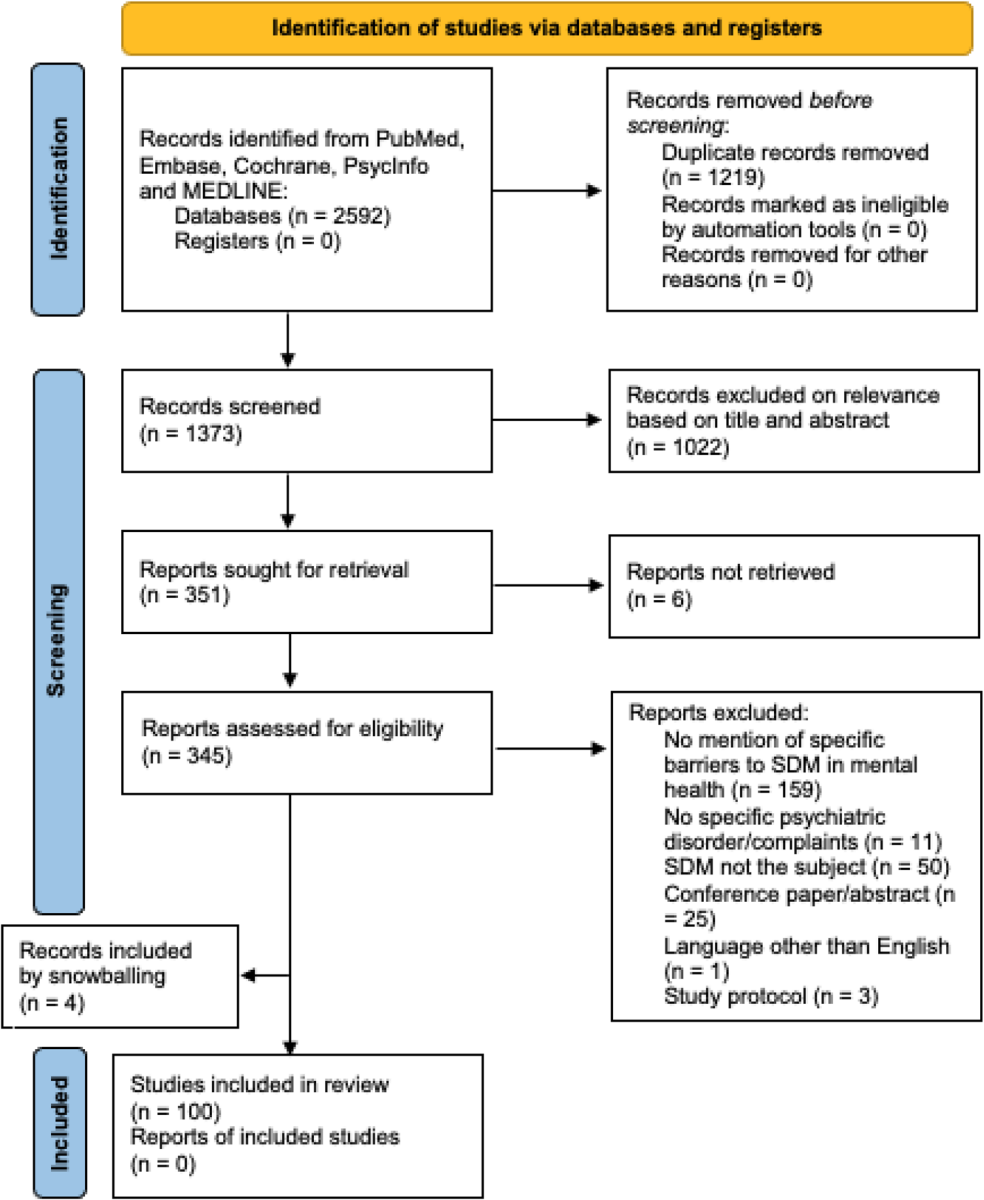
PRISMA flow diagram of the search strategy

We conducted a systematic search of the literature using the electronic databases PubMed, Embase, Cochrane, PsycINFO, and MEDLINE looking for publications using the terms (Shared decision making OR SDM) AND (psychiatr* OR mental) in their titles or abstracts. The search was performed in September 2021 and there were no limits for publication date. Papers were excluded when the language was other than English or if a full text was not available. There were no restrictions for patient variables such as age or diagnoses. The authors determined whether the papers explicitly addressed challenges of SDM in mental health practice, leaving 345 eligible articles that were read in their entirety and further discussed between the authors (see Fig. 1, PRISMA flow diagram). As our goal was to make a comprehensive inventory of all perceived barriers to SDM in clinical practice, we deliberately refrained from setting an inclusion threshold and included research data but also opinions, letters, and comments. In consensus, we categorised the barriers we identified using an elementary framework of micro-, meso- and macro-level themes.

## Results

We included 100 publications in our review (Appendix). We summarised all 20 barriers to SDM we identified into micro-, meso-, and macro-level themes presented in Table 1.

**Table 1:** Barriers to SDM implementation in mental health care per care-delivery level

Micro level	Meso level	Macro level
Decision incapacity	Unhelpful relations in inpatient settings	Stigmatisation
Disempowerment	Continuity of care	Absence of a practical model
Disease denial, discordant model of illness	Physical facilities	Cultural bias
Obligation to offer/choose the right treatment	Insufficient information, knowledge and decision aids	Lack of digital access/low level of education and literacy
Treatment adherence monitoring	Time constraints	Insurance policy/financial constraints
(History of) coercive treatment/restrictive measure	Adopting new roles in modern health-care visions	
Three-way communication	Working with standardised treatment protocols/guidelines	
Negative attitudes and countertransference		

## Barriers at the Micro Level: In the Consultation Room

### Decision Incapacity

Several core features of psychiatric disorders can impede SDM, such as the episodic course of the illness, where it depends on the current phase of the disorder whether patients are able to process information and be motivated to actively participate in decisions pertaining to their treatment (Beyene et al., 2018; Drivenes et al., 2019; Jeste et al., 2018; Jorgensen & Rendtorff, 2018; Kalsi et al., 2019; McCabe, 2017). Also in crisis situations, a patient’s decision capacity can be temporarily hampered (Farrelly et al., 2014; Wills, 2010).

Decisional incapacity (episodic or temporary) may play an important role in many psychiatric illnesses, particularly affecting patients with psychotic, depressed, or manic disorders, and patients with obsessive–compulsive disorder or psychogenic polydipsia (Brennan et al., 2019; de Las Cuevas et al., 2012; Guidry-Grimes, 2018; Stein Dan, 2017). In depression, core symptoms such as concentration problems or poor executive functioning can prompt a patient’s preference of a more passive decision-making style (Alguera-Lara et al., 2017; Butler et al., 2015; Curtis et al., 2010; Dahlqvist Jonsson et al., 2015; Fosgerau & Davidsen, 2014; Keij et al., 2021). In schizophrenia, inherent symptoms such as (chronic) suspicion and cognitive impairment can compromise the SDM process (Fosgerau & Davidsen, 2014; Huang et al., 2020a; Ishii et al., 2017), while other symptoms such as deficient social and communication skills or loss of self can make full engagement in SDM difficult (Curtis et al., 2010; Schauer et al., 2007; Storm & Edwards, 2013).

Finally, the stage of illness will likewise have an impact. In the advanced stages of dementia, for instance, consensus will no longer be achievable (de Las Cuevas et al., 2012).

### Disempowerment

Low self-esteem and a lack of empowerment, self-efficacy, and motivation for participation in decision making can result from psychiatric symptoms, causing helplessness and a further drop in motivation to actively engage in SDM. When patients are unsure about their ability to make decisions, the risk of their refraining from participating in the decision-making process is high (An et al., 2017; Deegan et al., 2017; Drake et al., 2010; Hamann et al., 2011; Huang et al., 2020a).

Patients indeed emphasized that their interest in and desire to engage in SDM were hampered by their negative depressive symptoms, which led them to adopt a more paternalistic decision-making attitude (Hamann et al., 2006, 2016). Additionally, Brooks and colleagues describe that cynicism on the part of both service users and service providers can hinder the SDM process (Brooks et al., 2019), with patients feeling disempowered, having the impression they are not part of any decision, or lacking a sense of ownership (Jorgensen & Rendtorff, 2018; Morán-Sánchez et al., 2019).

### Disease Denial and Discordant Models of Illness

SDM can be difficult when patients do not recognise or deny having a mental health problem or when patients and clinicians have very different opinions about the diagnosis or treatment plan, which may even cause either or both parties to refrain from or refuse to engage in SDM (Adams & Drake, 2006; Guidry-Grimes, 2020; Hamann et al., 2003; Morán-Sánchez et al., 2019). For example, one can imagine that someone with anorexia nervosa will not agree with gaining weight as a treatment goal or even refuse to see the weight loss as a problem or illness in the first place (Brennan et al., 2019).

Having a alternative explanatory model of illness is a similarly complicating factor: patients may perceive their symptoms as untreatable, something they cannot recover from, and/or attribute their symptoms to other factors such as life circumstances rather than to the disorder. Hence, it is critical that patient and clinician agree on the actual problems and goals at hand before any attempts at SDM are made (Patel et al., 2014).

### Obligations to Offer/Choose the Right Treatment

Both clients and health professionals mention (the feeling of) having obligations to society as an obstacle to SDM (Mahone et al., 2011a; Morant et al., 2016; Rogers et al., 1998), with clients specifying that this social control keeps them from being autonomous (Rogers et al., 1998).

Psychiatrists may avoid involving patients in decisions out of fear of the negative consequences the choice of the patient may have or because of their legal and moral obligations to make responsible treatment decisions (Beyene et al., 2018; Brophy et al., 2019; Guidry-Grimes, 2018; Gurtner et al., 2020; Hayes et al., 2019b; Moleman et al., 2020). Likewise, patients, including adolescents and their parents, convey that a fear of making the wrong decision negatively affects their involvement in the decision-making process (Hamann & Heres, 2014; Hamann et al., 2009, 2017b; Hayes et al., 2019a; Mahone et al., 2011b).

Both mental health practitioners and patients report a history of substance misuse as the reason for their preferring a more paternalistic approach to decision making (Huang et al., 2020a; Lukens et al., 2013; Slade, 2017). Clinicians tend to opt for this style when they fear that their client may otherwise relapse or turn or revert to (taking part in) criminal activities (Lukens et al., 2013).

### Treatment Adherence Monitoring

Improving treatment adherence can be one of the reasons for health professionals to continue trying to engage patients in the decisions concerning their treatment (Barr et al., 2016; Fisher et al., 2016; Harris et al., 2017; Jager et al., 2014; Kreyenbuhl et al., 2009; Patel et al., 2008; Younas et al., 2016). However, too much focus on treatment or medication adherence can induce discomfort in family members, whereby they feel that the problems of their loved one are being medicalised. Also, to be put in the role of “medication monitor” can create distrust between patient and family (Bradley & Green, 2017), specifically when there is a discrepancy between the self-reported medication adherence and the true situation (Ali et al., 2015; Patel et al., 2008). Conversely, some clinicians fear that engaging patients in decisions may lead to non-adherence to medication (Fox, 2021; Morant et al., 2016).

### (History of) Coercive Treatment /Restrictive Measures

A past or current compulsory treatment or hospital admission can have traumatic effects on patients, unintentionally demotivating them to cooperate in their recovery process and negatively affecting the doctor–patient relationship, which can challenge present and future treatment adherence (Brennan et al., 2019; Brooks et al., 2019; Drake, 2018; Drivenes et al., 2020; Giacco et al., 2018; Gurtner et al., 2020; Hamann & Heres, 2014; Hamann et al., 2011, 2015, 2016; Lovell et al., 2018; Mahone et al., 2011b; Morán-Sánchez et al., 2019; Morant et al., 2016; Nott et al., 2018; Quirk et al., 2012; Rogers et al., 1998; Sather et al., 2019).

Patients may have great difficulty showing any kind of vulnerability through open communication with their psychiatrist because of a history of abuse of power (Pavlo et al., 2019; Torrey & Drake, 2010). When they lack the ability to be honest and open in their communications, SDM is hardly possible (Smith & Williams, 2016). The fact that psychiatrists are legally permitted to use coercion, and some interventions may necessarily be coercive, can undermine the trust patients have in both their provider and the treatment (Angell & Bolden, 2015; Brooks et al., 2019; Curtis et al., 2010; Lin et al., 2020; Morán-Sánchez et al., 2019; Quirk et al., 2012; Torrey & Drake, 2010). In many cases, clinicians will do their utmost to try and inform patients and their family adequately without fully involving them in the decisions (Bradley & Green, 2017).

Psychiatrists also mention that the approach is not applicable in certain circumstances, mentioning acute or mandatory hospital admissions and situations in which involuntary treatment is legally permitted and deemed necessary, for instance when the patient’s behaviour indicates an intent to harm him/herself or another person (Brennan et al., 2019; Hamann & Heres, 2014; Lukens et al., 2013).^.^

### Three-Way Communications

Making decisions together with the patient and next of kin or carer is undoubtedly more challenging than it is in two-way communications (McCabe, 2017). Although clinicians acknowledge the potential of caregiver engagement, some family members/carers can be unhelpful or seek support for themselves, hampering carer–clinician interaction and cooperation (Schuster et al., 2021). The fear that including the third-party may make SDM more difficult, may lead to a lack of co-operation between the clinician and the family (Schuster et al., 2021). Preferences of the family member/parent may not be concordant with those of the (young) patient (Giacco et al., 2018; Langer & Jensen-Doss, 2016; Liverpool et al., 2020; Simmons et al., 2013), and parents may be over-emotional, negatively affecting their ability to evaluate the situation objectivity (Huang et al., 2021). Parental conflict can also preclude further involvement (Simmons et al., 2013). Especially when children are involved, a younger age was mentioned as a complicating factor for SDM: the developmental stage of the child can be in conflict with their legal age to decide (Simmons et al., 2013). In involuntary care situations, the involvement of family members is possibly even more challenging, where high levels of stress can compromise their decision-making capacity (Giacco et al., 2018), with some adopting non-helping behaviours, for instance by demanding specific care (Huang et al., 2021). All this may impinge on the patients’ self-confidence and autonomy (Huang et al., 2020a).

Like in two-way consultations, practical barriers to SDM such as inadequate provision of information, unhelpful staff, and poor communication also occur in three-way consultations, where stigmatisation of the (young) patient with the mental illness may also inhibit SDM contributions of third parties (Bradley & Green, 2017).

### Negative Attitudes, Misconceptions, and Countertransference

Common misconceptions about SDM can also hamper its successful application. There is the belief that patients will feel less supported if asked for their views or that the SDM process is (more) burdensome for them. Also patients would not wish to be, or cannot be, involved. There are also clinicians who claim “they are already using SDM” while the evidence shows the contrary (Brooks et al., 2019; Curtis et al., 2010; Farrelly et al., 2014; Hamann & Heres, 2014; Hopwood, 2020). Both patients and professionals describe cynicism or pessimism as being non-helpful attitudes (Brooks et al., 2019; Morant et al., 2016), with negative countertransference and clinicians’ therapeutic pessimism undermining their therapeutic capacity and thereby the SDM process (Guidry-Grimes, 2020).

If patients have preconceived opinions about mental health services and concerns about confidentiality, this can be a potential barrier to their disclosing information, precluding candid decision-making (Simmons & Hetrick, 2012).

## Barriers at the Meso Level: The Organisation of Care Services

### Unhelpful Relations in Inpatient Settings

Brennan et al. (2019) describe the issue of relying on information volunteered by or sought from other patients in case of eating disorders. Particularly in inpatient settings, this can generate unhelpful judgments, carrying the risk of decisions being more or solely based on other patients’ experiences, or engendering a sense of competition among fellow inpatients (Brennan et al., 2019).

### Continuity of Care

Discontinuity in the medical and nursing staff is a frequently described barrier to SDM as it negatively influences the doctor–patient relationship and may cause a lack of trust (Brooks et al., 2019; Gurtner et al., 2020). Such discontinuity also necessarily leads to a loss of information: previous decision-making talks (reflecting the decision process) or (one-sided or joint) decisions may be unknown to the new staff (Hayes et al., 2019a). The implementation of newly trained communication strategies commonly fails because of a fragmented health care delivery and shortness of staff (Brooks et al., 2019). Shortage of staff can lead to rushed, more paternalistic consultations, and lack of time to rethink decisions at a later stage (Hayes et al., 2019a; Lin et al., 2020; Morant et al., 2016), with both patients and caregivers describing these difficulties (Hayes et al., 2019b).

Discontinuity of care may also result from patients moving to different service providers, for instance when a patient transits from an inpatient setting to a community-based facility or from primary to specialist care, where it can be confusing for the patient when different caregivers have different views (Morant et al., 2016). Continuity of care is of great importance in long-term mental health care (Drivenes et al., 2019), which necessarily relies on a good exchange of all relevant patient information.

### Physical Facilities

Not mentioned often but a very logical factor for successful SDM are the physical properties of the treatment setting. If they do not support a healthy environment for listening and talking this will inevitably affect the quality of the process. Noisy and busy wards are evidently not helpful for SDM, as is the lack of a private, quiet space to sit down together (Giacco et al., 2018). The architecture of buildings or wards may originally have been developed with other needs in mind, where we now demand different arrangements and amenities, such as when wards that were designed for the medical treatment of adults are now being used for the mental health management of young people (Hayes et al., 2019a).

### Insufficient Information, Knowledge, and Decision Aids

The many treatment options available in mental health care frequently stand in the way of using SDM (Deegan, 2010; Fisher et al., 2016; Harris et al., 2017; Matthias et al., 2012; Wolpert et al., 2017), but this may also apply when options are limited (Rodenburg-Vandenbussche et al., 2020). This latter holds, for instance, for the treatment of eating disorders or psychotherapy for children and adolescents (Brennan et al., 2019; Hayes et al., 2019b; Langer & Jensen-Doss, 2016). Because clinicians are generally pushed for time, and because of often limited access to research bases, they cannot always keep up with (recommendations on) the latest treatment options, while conveying information about the diagnosis and prognoses of different treatments can be complex, where clinicians sometimes resort to discussing less treatment options than actual possible (Hayes et al., 2019a; Langer & Jensen-Doss, 2016; Mahone et al., 2011b).

Service users report they receive too little information, mostly regarding their medication. Most of the time the information is provided verbally by the treating clinician without the aid of any decision support tools. There is a lack of quality support tools (Kalsi et al., 2019) and the decision aids that do exist for use in mental health care are sometimes provided by the treatment manufacturers and may thus be biased. The decision aids are aiming at mild to moderate psychiatric diseases and not very specific on all existing treatment options. Most of the patients are not properly explained the pros and cons of the medication to be taken (Curtis et al., 2010).

### Time Constraints

As already alluded to above, a frequently mentioned obstacle to SDM is that the method costs too much time, what with health professionals already being under time pressure in their consultations, with the looming risk of unvoiced patient agendas (Alguera-Lara et al., 2017; Ali et al., 2015; Aoki, 2020; Deegan, 2010; Drake et al., 2009; Hamann et al., 2015; Hayes et al., 2019a; Hopwood, 2020; Huang et al., 2020a, 2021; Malpass et al., 2010; Matthias et al., 2012; Milte et al., 2015; Morant et al., 2016; Schön et al., 2018; Simmons et al., 2013; Torrey & Drake, 2010; Younas et al., 2016). It is interesting that, with lack of time being a much heard argument against SDM, a recent Cochrane review of SDM interventions in mental health care found no evidence that SDM has any effect on the total length of the consultation (Aoki et al., 2022), suggesting that insufficient time is most likely a perceived rather than an objective barrier.

Another related common misconception is the belief that sharing decisions with, for example, a depressive patient is not a good use of time. Physicians tent to ‘treat first and involve patients later’ (Hopwood, 2020). Also, time constraints can prevent health professionals from attending training courses to improve the implementation of SDM (Brooks et al., 2019; Schön et al., 2018). Patients also complain of limited time resources, stating that they felt treating staff were too busy (Barnett et al., 2021; Curtis et al., 2010; Hamann et al., 2015; Hopwood, 2020; Lin et al., 2020; Morant et al., 2016; Pappa et al., 2021).

### Adopting New Roles Consistent with Current Health Policies

The shift in power SDM requires can challenge the authority and autonomy of the clinician, where it is not uncommon that the ultimate decision is still made by the doctor. Both clinician and patient have to develop skills to adapt to their new roles, while also changes at the organisational level may be necessary (Economou et al., 2019; Hamann & Heres, 2014; Hopwood, 2020). Mutual misconceptions about the stakeholders’ roles in SDM can lead to “unvoiced agendas” or unrealistic expectations (Malpass et al., 2010; Verwijmeren & Grootens, 2018). Mental health carers and patients both feel that the long history of paternalistic decision-making may drive the preference of patients not to be involved in SDM (Huang et al., 2021; Jorgensen & Rendtorff, 2018; Lin et al., 2020; Slade, 2017).

Slade (2017) highlights the importance of institutional structures that “can powerfully socialize a patient into a moral duty to be treatment-adherent” (Slade, 2017). A traditional “asylum-based” health care system may contribute to a micro-culture where decision-making is more prominently clinician-led.

Mental health care delivery is defined by short-term interventions and aversion of risks, in which relapse prevention is deemed more important than the potential side effects of long-term medication use. Most often, health professionals rely on medication as the treatment of choice instead of opting for non-pharmacological treatments (Morant et al., 2016). Owing to this pragmatic and paternalistic organisational culture, having staff adopt new communication models and attend training courses can be challenging (Brooks et al., 2019).

### Treatment Protocols/Guidelines

Patients can find SDM cumbersome when their health professional sticks to set procedures and checklists, which appears to them to conflict with a person-centred approach (Beyene et al., 2018), where adhering to guidelines will often result in patients being offered a single recommended treatment option. On the other hand, ignoring guidelines can result in insufficient or inadequate care and consequential increases in medical costs (Moleman et al., 2020).

## Barriers at the Macro Level: Socioeconomic Factors

### Stigmatisation

In mental health both consumers and providers perceive self-stigma, shame, prejudice, and discrimination to be common barriers for SDM (Alguera-Lara et al., 2017; Aoki, 2020; Brophy et al., 2019; Curtis et al., 2010; Dahlqvist Jonsson et al., 2015; Farooq et al., 2017; Guidry-Grimes, 2020; Huang et al., 2020a, 2021, Moleman et al., 2020; Patel et al., 2014; Simmons et al., 2013; Slade, 2017), especially in relation to medication (Fosgerau & Davidsen, 2014) and not only in adult but also in child mental health care (Butler, 2014; Butler et al., 2015). Also, stigma may reinforce existing reservations from professionals to include family members in decision making (Bradley & Green, 2017; Huang et al., 2021). Hamman et al. (2017) describe the “why try” effect in patients with high self-stigma and shame, which can lead to adverse outcomes, reduced help and information seeking, and poor treatment adherence (Hamann et al., 2017a). Mental health professionals furthermore anticipate that the majority of their patients might not feel free to be completely open and honest, possibly out of fear that no-one will understand their thoughts (Mahone et al., 2011b). This self-stigmatisation is exacerbated when providers put into perspective or lower their patients’ expectations of employment, individual growth, education, and housing by referring to poor prognoses and outcomes (Schauer et al., 2007). This may cause patients to become stuck in a vicious cycle, with self-stigma preventing them from engaging in SDM, prompting their preference for a more paternalistic decision style, which, in turn, may fuel their self-stigma (Hamann et al., 2017a).

### Absence of a Practical Model

The ethics and moral imperatives of a shared decision approach are clear for most clinicians, but they often find its practical implementation challenging, requiring all kinds of organisational and cultural shifts (Brooks et al., 2019; Lovell et al., 2018). The absence of a universal implementation model may be one of the reasons why SDM is not readily adopted by mental health professionals (Gurtner et al., 2020).

### Cultural Bias

The concept of SDM stems from a Western, liberal, individualistic view of human relations. Note that with a few exceptions the literature on SDM derives from Western countries. In other cultures, a more paternalistic approach to care—or even a culture of obedience to authority—is the norm, as are different traditions in care delivery or attitudes with respect to individual decisions (Huang et al., 2020b, 2021; Lin et al., 2020), which cultural differences become more notable with societies becoming increasingly more multicultural. Where perceptions of health problems differ widely or where providers and consumers do not (literally or figuratively) speak the same language, and in the absence of decision aids tuned to different cultures, the practice of SDM is complicated or even no option (Giuliani et al., 2020).

### Lack of Digital Access/Low Level of Education and Literacy

Limited access to computers and Internet services, or a lack of computer experience may complicate SDM for patients (Morán-Sánchez et al., 2019), with many decision aids facilitating SDM requiring some degree of computer literacy (Andrews et al., 2010; Grim et al., 2017). No or limited access to the Internet deprives patients of valuable sources of information such as patient information websites and online fora that can foster their information position. In some countries patients have online access to their full medical records, while in others the computer infrastructure needed is inadequate or even lacking (Drake et al., 2009, 2010).

Making decision and processing information in general requires basic literacy skills. People with reduced cognitive or mental abilities, a lack of formal education or training, or an immigration history and/or poor foreign language skills will always be lagging in this respect, complicating or precluding a (full) sharing of decisions (Ali et al., 2015; Gurtner et al., 2020; Mahone et al., 2011b).

### Insurance Policy/Financial Constraints

SDM is also hampered when the choice of treatment options (e.g. medication) is restricted by health insurance policies, primary care trusts, or when local guidelines are affected by budget cuts (Mahone et al., 2011a; Shepherd et al., 2014). Some patients will consequently be limited in their choice of available treatments since they have insufficient coverage or money to pay for a specific therapy (Huang et al., 2020b).

## Discussion and Conclusion

### Discussion

Efforts to apply shared decision making in mental health services uncovered practical difficulties and challenges, conceivably causing some hesitancy in its wider implementation. Seeking to paint a broader picture of the objective and subjective barriers to its use in our field, we took a narrative approach to the literature, evaluating all levels of evidence. To the best of our knowledge, we are the first to have done so.

By categorising all reported barriers to SDM thematically, we feel we have provided a comprehensive overview of current views on its challenges, while enabling us to identify possibilities for change that will help foster and improve the implementation and research of SDM in the mental health setting. Some hurdles may derive from mistaken beliefs, some may be temporary, and some will likely be addressed in the near future (e.g. providing decision aids for specific medical/psychiatric conditions), while others will continuously have to be navigated because they are fundamental to the complexity and dynamics of mental health care. We will conclude our review by presenting four perspectives on SDM in psychiatry based on this overview and our experience as treating clinicians and researchers in this field.

### Practice Implications

#### Cultivating Long-Term Therapeutic Relationships

In mental health in general but especially in patients with chronic and severe psychiatric disorders, SDM will never involve a single or simple decision, it’s a dynamic construct. The process depends on the longer-term course of the disorder, with prognostic uncertainties, changing symptoms, complex relations, and a whole range of different decisions needing to be made. Patients’ wishes and values can evolve during the course of the treatment. For patients, the process may feel as a long drawn-out or never-ending journey, where they will need a guide on whom they can rely. This asks for a time-contingent approach, with frequent consultations that are not restricted to crisis situations but should also be scheduled in symptom-free intervals to inform and motivate the patient, check and update past decisions, and to avoid loss of connection, non-adherence to treatment, or dropout (Grootens & Verwijmeren, 2023). If we acknowledge that mental health care is a joint, continuous, and sometimes arduous journey, we will discover the true therapeutic power and effectiveness of SDM in psychiatry.

#### Sharing Risks and Responsibility While Allowing for Moral Obligation

One of the most prominent characteristics of severe psychiatric syndromes is their impact on decisional capacity, with the (temporary) impairment sometimes obliging clinicians to overrule the patient’s wishes. In mental health and particularly in the context of SDM, monitoring cognitive and decisional control, and assessing risks are important aspects of a psychiatrist’s tasks, where coercion to prevent self-harm or harm to others can be fundamental. As risks are inevitable in the care of severe mental health patients, the concept of “shared risk taking” has been suggested (Zisman-Ilani et al., 2021a). SDM thus also means shared responsibility: not only the decision is shared but also the responsibility that comes with the decision. All members of the triad (patient, next of kin, and health professional) will face moral dilemmas in the decision process, sometimes having to decide which ethical principal should prevail (e.g. autonomy vs non-maleficence if tapering off of medication is being considered). We always need to be aware that every mental health issue has unique aspects that differ for every patient, making moral deliberation imperative in many cases to thus arrive at the best-possible decision, be it consensual or coerced.

#### Boundaries of SDM

The many different definitions of SDM found in literature (Stiggelbout et al., 2015) can be seen as a barrier itself. While some authors suggest that SDM can *only* take place when the professional is in equipoise about the options (SAMHSA, 2010), we have used a broader definition which also includes decisions that were initially not preferred by the clinician or the patient, as long as both parties agree with final outcome at the end of the decision process. There is a fine line between SDM and motivational interviewing (Elwyn et al., 2014) in mental health care. There can be a grey area between ‘fully agree’ via ‘tolerate’ and ‘neutral’ to ‘not agree’, and providers must take clear, honest and transparent positions if they think a shared approach is not within reach.

#### Promoting Inclusion and Diversity

As sharing decisions is an empowering process, it will improve a patient’s self-esteem and self-efficacy (Huang et al., 2020b). Preconceptions of SDM with patients with a psychiatric diagnosis and (counter)transference issues include a presumed impairment in reasoning, especially in patients coping with personality disorders (Guidry-Grimes, 2020). Mental health professionals, patients, and next of kin or caregivers, however, do not operate in a vacuum; they are part of a society in which, unfortunately, stigmatisation is still common. Worldwide, there is much talk of inclusion and diversity and these themes are just as topical for people with mental health problems. We need to continue to work hard at eradicating *all* stigma and self-stigma.

### Conclusion

Our inventory of barriers to SDM in mental health care shows how challenging its implementation is perceived to be. Although we feel many of these obstacles can be overcome in the course of the implementation process, some challenges are inherent to the nature of mental health care, with its long-term patient–clinician relationships, complex dynamics, moral dilemmas and social implications. While SDM remains the moral imperative (Drake & Deegan, 2009), at the same time we need to acknowledge the clinical reality that (a more) paternalistic approach is sometimes opportune or even crucial in order to secure the best care possible. If we view SDM as a continuum, we can allow for different patients and variable situations, where sharing can at some point encompass most or some care decisions or where sharing may need to be confined to explaining urgently needed actions. In this continuum, let us try to make care ‘as shared as possible’.

## Appendix

Main characteristics of the publications reviewed

**Table:** 

Author	Publication year	Study design	Participants
Adams and Drake	2006	Narrative review	n.a.^a^
Alguera-Lara et al.	2017	Narrative review	18 publications
Ali et al.	2015	Cross-sectional, self-reported survey	29 psychiatric providers
An et al.	2017	Quasi-experiment with a non-equivalent control group pre/post-test design	60 patients with schizophrenia or schizoaffective disorder
Andrews	2010	Applied research	n.a.
Angell and Bolden	2015	Qualitative study	36 psychiatrist-patient/client conversations
Aoki	2020	Conceptual review	70 articles
Aoki	2022	Cochrane systematic review	15 RCTs^b^
Barnett	2021	Qualitative study	100 patients (nos) and 35 prescribers
Barr et al.	2016	Online cross-sectional study	972 patients with depression and 244 clinicians
Beyene	2018	Qualitative study	8 mental health care professionals
Bradley	2017	Qualitative study	46 family members of patients with SMI^c^ 55 staff members
Brennan	2019	Narrative review	n.a.
Brooks	2019	Longitudinal qualitative study	21 professionals, 29 service users and 4 carers in CMH^d^
Brophy	2019	Qualitative study	29 consumers with SMI, 30 family members, 10 psychiatrists, 20 MHPs^e^
Butler	2014	Cross-sectional study	52 parents of children aged 2–7 years receiving primary mental health care
Butler et al.	2015	Cross-sectional study	21,721 parents of children aged 2–17 years with mental-health and physical conditions
De las Cuevas et al.	2012	Cross-sectional study	100 psychiatrists and 125 psychiatry registrars
Curtis et al.	2010	Narrative review	n.a.
Dahlqvist-Jönsson et al.	2015	Cross-sectional study	20 patients with SMI
Deegan	2010	Narrative review	n.a.
Deegan et al.	2017	Cross-sectional study	17,385 clients with SMI
Drake et al.	2009	Narrative review	n.a.
Drake	2018	Letter from the author	n.a.
Drake et al.	2010	Narrative review	n.a.
Drivenes	2019	Cross-sectional study	60 medical doctors, 97 psychologists, 127 with a degree in nursing, social science or pedagogy
Drivenes	2020	Cross-sectional study	992 patients with a psychiatric diagnosis, 267 therapists
Economou	2019	Letter to the editor	n.a.
Farooq et al.	2017	Cochrane systematic review	0 articles
Farrelly	2014	Mixed methods thematic analysis	221 service users with a psychotic disorder
Fisher et al.	2016	Systematic review	13 articles
Fosgerau and Davidsen	2014	Cross-sectional study	12 GPs^f^ and 10 psychiatrists
Fox	2021	Autoethnography	n.a.
Giacco	2018	Qualitative study	22 patients and 16 clinicians in involuntary mental health care
Giuliani	2020	Literature review	n.a.
Grim et al.	2017	Mixed-methods study	A total of 75 participants in different phases of the study
Guidry-Grimes	2018	Dissertation on a philosophical investigation	n.a.
Guidry Grimes	2020	View from the author	n.a.
Gurtner	2020	Integrative review	14 articles
Hamann and Heres	2014	Applied research	n.a.
Hamann and Heres	2015	Letter to the editor	n.a.
Hamann, Bühner et al.	2017	Cross-sectional study	329 individuals with a schizophrenia spectrum disorder
Hamannn et al.	2017	Study protocol	n.a.
Hamann et al.	2016	Qualitative study	16 patients with psychotic and depressive disorders and 21 physicians
Hamann et al.	2006	Randomized controlled trial	107 patients with schizophrenia
Hamann et al.	2003	Narrative review	4 studies
Hamann et al.	2009	Cross-sectional study	352 psychiatrists
Hamann et al.	2011	Cross-sectional study	101 patients with schizophrenia and 102 patients with multiple sclerosis
Harris et al.	2017	Qualitative study	9 patients with antipsychotic medication, 11 carers, 10 consultant psychiatrists, two CPNs^g^ and one pharmacist
Hayes	2019	Qualitative study	9 young people with internalising difficulties and 10 parents
Hayes	2019	Qualitative study	15 clinicians in child and youth mental health care
Hopwood	2020	Review article	n.a.
Huang	2020	Integrative review	46 articles
Huang	2020	Qualitative study	12 inpatients with schizophrenia
Huang	2021	Qualitative study	10 psychiatrists and 23 mental health nurses
Ishii et al.	2017	Randomized clinical trial	24 patients with schizophrenia
Jager et al.	2014	Longitudinal prospective cohort study	211 adolescents receiving psychosocial care
Jeste	2018	Overview of literature	n.a.
Jorgensen	2018	Integrative review	7 articles
Kalsi	2019	Review article	n.a.
Keij	2021	Qualitative study	15 patients and 16 professionals in different specialities
Kreyenbuhl et al.	2009	Narrative review	n.a.
Langer	2016	Conceptual review	n.a.
Lin	2020	Qualitative study	20 patients with mental illness residing in halfway houses
Liverpool	2020	Scoping review	31 articles
Lovell	2018	Pragmatic cluster randomised trial	604 patients with mental illness and 90 carers
Lukens et al.	2013	Randomized factorial survey	87 social workers of adults with SMI
Mahone et al.	2011	Qualitative study	7 focus groups with different stakeholder groups
Mahone et al.	2011	Qualitative study	4 family members, 4 prescribers, 6 other caregivers, 24 patients of a local mental health clinic from focus groups
Malpass et al.	2010	Qualitative study	10 pairs of GPs and patients with a depressive episode
Matthias et al.	2012	Qualitative study	40 patients with SMI
McCabe et al.	2017	Letter from the author	n.a.
Milte et al.	2015	Cross-sectional study	59 family meetings involving geriatricians, patients and caregivers
Moleman	2020	Qualitative study	68 healthcare professionals from different specialities and 15 patients
Moran-Sanchez	2019	Mixed methods study	107 patients with bipolar disorder or schizophrenia and 100 non-psychiatric comparison subjects
Morant et al.	2016	Conceptual review	n.a.
Nott	2018	Mixed methods study	109 patients with mental illness
Pappa	2021	Qualitative study	100 services users in community mental health teams and 35 prescribers
Patel et al.	2008	Systematic review	24 articles
Patel et al.	2014	Qualitative study	15 MHPs
Pavlo	2019	Mixed methods study	n.a.
Quirk et al.	2012	Qualitative study	9 psychiatrists
Rodenburg-Vandenbussche	2020	Qualitative study	17 outpatients with depression, anxiety and OCD^h^ and 33 clinicians
Rogers et al.	1998	Qualitative study	34 patients with schizophrenia or schizoaffective disorder and long-term neuroleptic medication
Sather	2019	Qualitative study	10 former patients with mental health problems
Schauer et al.	2007	Narrative review	n.a.
Schon	2018	Mixed methods process evaluation study	95 MHPs
Schuster	2021	Cross-sectional study	94 triads of service users, their caregivers and clinicians in inpatient mental health care
Shepherd	2014	Qualitative study	26 psychiatrists
Simmons	2012	Commentary	n.a.
Simmons et al.	2013	Qualitative study	22 health professionals
Slade	2017	Narrative review	n.a.
Smith and Williams	2016	Narrative review	n.a.
Stein Dan	2017	Letter from the author	n.a.
Storm and Edwards	2013	Narrative review	n.a.
Torrey and Drake	2010	Narrative review	n.a.
Verwijmeren and Grootens	2018	Qualitative study	82 patients with bipolar disorder, 6 health professionals
Wills	2010	Letter from the author	n.a.
Wolpert et al.	2017	Narrative review	n.a.
Younas et al.	2016	Qualitative study	13 patients

^a^Not applicable

^b^Randomised controlled trial

^c^Severe mental illness

^d^Community mental health

^e^Mental health practitioners

^f^General practitioners

^g^Community psychiatric nurses

^h^Obsessive–compulsive disorder

## Data Availability

All data and materials support the published claims and comply with field standards.
